# Functions of PARylation in DNA Damage Repair Pathways

**DOI:** 10.1016/j.gpb.2016.05.001

**Published:** 2016-05-27

**Authors:** Huiting Wei, Xiaochun Yu

**Affiliations:** 1 Department of Immunology, Tianjin Key Laboratory of Cellular and Molecular Immunology, MOE Key Laboratory of Immune Microenvironment and Disease, School of Basic Medical Sciences, Tianjin Medical University, Tianjin 300070, China; 2 Department of Cancer Genetics and Epigenetics, Beckman Research Institute, City of Hope Medical Center, Duarte, CA 91010, USA

**Keywords:** Poly ADP-ribosylation, PARPs, DNA damage response, PAR-binding modules, Ubiquitination

## Abstract

Protein **poly ADP-ribosylation** (PARylation) is a widespread post-translational modification at DNA lesions, which is catalyzed by poly(ADP-ribose) polymerases (**PARPs**). This modification regulates a number of biological processes including chromatin reorganization, **DNA damage response** (DDR), transcriptional regulation, apoptosis, and mitosis. PARP1, functioning as a DNA damage sensor, can be activated by DNA lesions, forming PAR chains that serve as a docking platform for DNA repair factors with high biochemical complexity. Here, we highlight molecular insights into PARylation recognition, the expanding role of PARylation in DDR pathways, and the functional interaction between PARylation and **ubiquitination**, which will offer us a better understanding of the biological roles of this unique post-translational modification.

## Introduction

Throughout the biological life, genomic stability of the organisms is always challenged by both endogenous and exogenous toxic stresses [1], [2]. It has been estimated that every cell could experience up to 10^5^ spontaneous DNA lesions per day [3]. To maintain genomic integrity, the organisms have evolved a series of sophisticated and precise mechanisms to protect their genome against the deleterious lesions, including cell cycle checkpoint, diverse DNA repair signaling pathways, chromatin reorganization, and protein modifications [4]. Among these responses, poly ADP-ribosylation (PARylation) is a pivotal post-translational protein modification (PTM) that appears rapidly at DNA damage sites [5], [6].

In human, ADP-ribosylation is catalyzed by poly(ADP-ribose) polymerases (PARPs), which consists of 17 members [7], [8], [9], [10]. PARPs primarily covalently attach the ADP-ribose (ADPR) unit via an ester bond to the carboxyl group of acidic residues such as glutamate or aspartate residues on the target proteins [11], [12], but cysteine (Cys) and lysine (Lys) residues could also act as acceptors [13], [14]. However, most of them are only able to transfer single mono(ADP-ribose) (MAR) group onto their target proteins [5], [15]. To date, PARP1, 2, and 3 have been identified to catalyze PARylation during DNA damage response (DDR) [5], [15]. In addition, tankyrases including tankyrase-1 (PARP5a) and tankyrase-2 (PARP5b) have also been shown to contribute to genomic stability [15], [16]. Among these PARPs, PARP1 is the founding member of PARP family for the synthesis of PAR chains. The mechanism of PARP1 activation by single-strand and double-strand DNA breaks (SSBs and DSBs) is well established [17]. Using NAD^+^ as substrate, PARPs repeatedly catalyze the transfer of successive units of ADPR moieties via a unique 2′,1″-*O*-glycosidic ribose-ribose bond to target proteins, finally producing PAR chain [5]. Several reports have demonstrated that PAR chains can comprise up to 200 ADPR units in length [5], [11], [17]. In addition, PARP1 can introduce branching into PAR chains through the 2″,1‴-glycosidic bond [18], [19].

In cells, PAR polymers are primarily degraded by PAR glycohydrolase (PARG), which possesses both exoglycosidic activity and endoglycosidic activity [20], [21], [22]. PARG efficiently cleaves the unique 2′,1″-glycosidic ribose-ribose bonds of the PAR chains and releases the free ADPR moieties [22], [23]. In addition, ADP-ribosylhydrolase 3 (ARH3) also exhibits the PAR-degrading activity, although ARH3 has only exoglycosidase activity [24], [25]. Neither PARG nor ARH3 can hydrolyze the proximal protein-bound ADPR unit from a PAR chain, possibly due to steric hindrance, thus leaving a MARylated protein. MARylated proteins can be recognized by different protein domain and thus serve as scaffolds for recruitment of proteins during diverse biological processes [22], [24]. Interestingly, a set of Macro domain-containing (MacroD) proteins have been found to exhibit hydrolase activities. These include the terminal ADPR protein glycohydrolase (TARG1/C6orf130) [26], as well as MacroD1 and MacroD2 [27], [28], [29], [30]. Earlier studies showed that these three enzymes can hydrolyze *O*-acetyl-ADPR, a metabolite derived from NAD during sirtuin2-catalyzed protein deacetylation, regulating diverse biological processes [31]. Recently, TARG, MacroD1, and MacroD2 were identified for their action in removal of glutamate-specific ADPR [26], [28], [29]. The hydrolysis of last ADPR from modified protein is the final and rate-limiting step of PAR chain degradation [32]. Like many other PTMs, synthesis and degradation of PAR chains is tightly and dynamically controlled *in vivo* with the half-life of only several minutes [4], [27]. If PAR chains cannot be hydrolyzed in a timely manner, excessive protein-free PAR chains can induce the apoptosis-like cell death, termed parthanatos [4], [27]. Parthanatos is another form of programed cell death which is distinct from necrosis and apoptosis. As a distinct death pathway, parthanatos is associated with PARP-1. The synthesis and accumulation of PAR chain will result in mitochondrial depolarization and nuclear apoptosis inducing factor (AIF) translocation, thus inducing cell death [33].

## PARylation and DNA repair pathway

### PARylation in base excision repair/SSB repair process

The base excision repair/SSB repair process (BER/SSBR) is a pivotal DNA repair signal pathway to repair oxidized bases, apurinic/apyrimidinic sites (AP sites, also known as abasic sites) or SSBs [1]. In cells, many chemical alterations such as oxidation, methylation, deamination, and hydroxylation can induce base damage and SSBs [1]. In the BER process, damaged bases are cleaved by DNA glycosylases, producing abasic sites, which are next processed by AP endonuclease (APE) into SSBs [2], [34]. These sites are further repaired through two different pathways termed short-patch repair and long-patch repair, which are distinct in terms of patch sizes and DNA repair factors involved [35].

**Figure 1:**
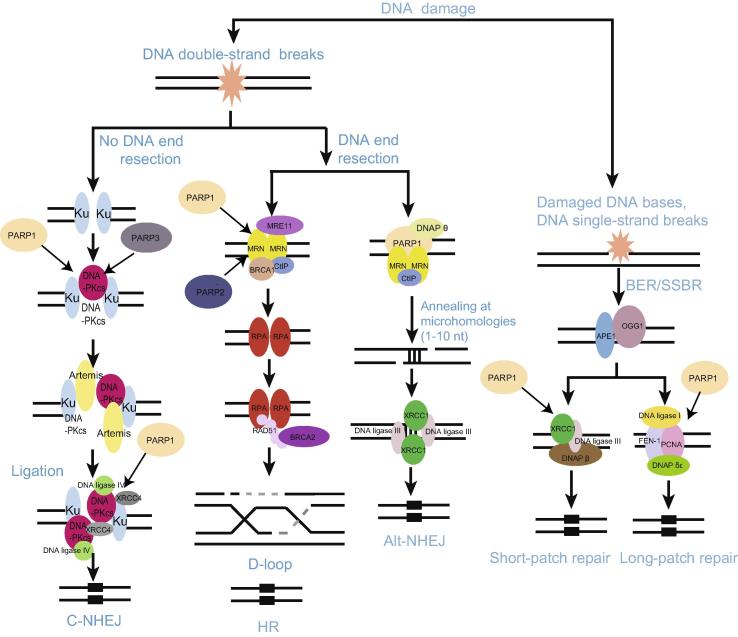
**PARylation mediates DNA damage repair** The scheme depicts DNA repair networks regulated by PARylation. As might be expected, PARPs interact physically and functionally with several DNA damage factors to promote their recruitment to the sites of DNA damage. alt-NHEJ, alternative non-homologous end joining; APE, apurinic/apyrimidinic (AP) endonuclease; BER, base excision repair; BRCA1, breast cancer type 1 susceptibility protein; BRCA2, breast cancer type 2 susceptibility protein; C-NHEJ, classical non-homologous end joining; CtIP, C-terminal-binding protein (CtBP)-interacting protein; DNAP, DNA polymerase; DNA-PKcs, DNA-dependent protein kinase, catalytic subunit; FEN-1, flap structure-specific endonuclease 1; HR, homologous recombination; MRE11, meiotic recombination 11 homolog 1; MRN, Mre11-Rad50-Nbs1 complex; OGG-1, 8-oxoguanine glycosylase 1; PARP, poly(ADP-ribose) polymerase; PCNA, proliferating cell nuclear antigen; RPA, replication protein A; SSBR, single-strand break repair; XRCC4, X-ray repair cross-complementing protein 4.

PARP1 can physically and functionally interact with SSBR factor X-ray repair cross-complementing protein 1 (XRCC1), which plays a major role in SSBR signal pathway, facilitating the recruitment and assembly of the SSBR machinery [35]. Our recent study indicates that the BRCA1 C Terminus (BRCT) domain of XRCC1 directly binds to PAR chain and mediates early recruitment of XRCC1 to DNA lesions [36]. Several reports have also demonstrated that PARP1 is able to interact with key factors of the BER/SSBR process including the DNA glycosylase 8-oxoguanine glycosylase 1 (OGG1), XRCC1, DNA polymerase (DNAP) β, DNA ligase III, proliferating cell nuclear antigen (PCNA), aprataxin, and condensin I [37], [38], [39], [40]. Many of these factors can undergo PARylation by PARP1 (Figure 1). Additionally, PARP2 has also been identified to interact with BER/SSBR proteins such as XRCC1, DNAP β, and DNA ligase III [41]. These findings support that PAR chain could provide a landing platform for the recruitment of DNA repair complexes as proposed by Masson et al. in 1998 [42].

### PARylation in DSB repair

DNA DSBs are regarded as the most detrimental DNA damage, which seriously and directly threaten genomic stability via interrupting the physical continuity of the chromosome [1]. The failure to repair DSBs will lead to catastrophic consequences such as oncogenesis, cell death, and developmental disorders [1]. To deal with DSBs, organisms have employed three major DNA repair mechanisms including classical non-homologous end joining (C-NHEJ), alternative non-homologous end joining (alt-NHEJ), and homologous recombination (HR). The choice of DNA repair pathway depends on whether the damaged DNA end is resected, which is likely mediated by the Mre11/Rad50/Nbs1 (MRN) complex and C-terminal-binding protein (CtBP)-interacting protein (CtIP). Once DNA resection is impeded, repair by C-NHEJ is invoked. However, if resection has occurred, HR and alt-NHEJ may compete with each other to repair the damaged DNA. RAD51 forms a filament at the site of SSB that drives strand exchange and facilities HR, whereas PARP1 may serve as a platform for recruiting alt-NHEJ repair factors such as DNAP θ [43].

### PARylation in C-NHEJ

Eukaryocytes mainly employ C-NHEJ to repair damaged DNA. The process is DNA end resection-independent, and is also unrelated to sequence homology. Therefore, C-NHEJ occurs throughout the cell cycle, but predominantly in G0/G1 and G2 phase [44], [45]. In the process of C-NHEJ, the Ku70/Ku80 heterodimer is recruited to DNA damage sites followed by loading of DNA-dependent protein kinase catalytic subunit (DNA-PKcs). Meanwhile, Ku70/Ku80 heterodimer facilitates the activation of the DNA ligase IV/XRCC4 complex. Accessory factors such as nuclease Artemis, aprataxin-polynucleotide kinase-like factor (APLF), or polynucleotide kinase/phosphatase (PNKP) process the damaged DNA end to be compatible for ligation. At the final step, the activated DNA ligase IV and its cofactor XRCC4, or Cernunnos/XRCC4-like factor (XLF), rejoin the DNA ends [46].

Several studies support an important role of PARP1 in C-NHEJ. Interaction between PARP1 and DNA-PKcs facilitates genomic integrity during V(D)J recombination and prevents tumor development [47]. It is of note that PARP can stimulate DNA-PKcs activity via PARylation *in vitr*o [48]. This interaction is further supported by *in vivo* evidence as reported recently. A structural PARP1/DNA-PKcs/Ku molecular complex has been identified in which PARP1 elicits a major architectural rearrangement of the DNA-PKcs-mediated synapsis [49]. Moreover, previous studies from our lab have shown that the BRCT domain of DNA ligase IV directly recognizes the ADP-ribose of PAR chains, which mediates the early recruitment of the ligase to DNA lesions. Such efficient recruitment may facilitate C-NHEJ [50].

### PARylation in alt-NHEJ

As a new DSB repair signal pathway, alt-NHEJ has attracted much attention recently [46]. When classical C-NHEJ is deficient, alt-NHEJ can be initiated by resected DNA end. Compared with C-NHEJ, alt-NHEJ is characterized by the following features: initiated by damaged DNA end resection; independent of the Ku70/Ku80 heterodimer, XRCC4, and DNA ligase IV; using complementary microhomologies—short stretches (1–10 nucleotides) that can anneal, to guide DNA repair and much less faithful than C-NHEJ [51]. PARP-1, XRCC1, DNA ligase III, PNKP, WRN, CtIP, NBS1, and ERCC1 have all been implicated in alt-NHEJ [46]. PARPs play pivotal roles in this process. PARP1 can recognize the broken DNA ends and create a scaffold for the recruitment of other DNA damage factors involved in alt-NHEJ. Finally, end-rejoining is carried out by the DNA ligase III/XRCC1 complex in coordination with PARP1 [52] (Figure 1). In addition, both XRCC1 and PNKP can be recruited to the DNA damage sites through PAR binding, which could occur at the early steps of alt-NHEJ [50].

### PARylation in HR

HR can be activated by single-stranded DNA (ssDNA) resection. The process produces a lagging strand gap or 3′overhang, which is the key step for HR [53]. Owing to its requirement for a sister chromatid, HR predominates in S and G2 phases, when the amount of DNA replication is highest and the sister template is available [45], [54]. HR is typically characterized by error-free [1], [55]. Using homologous sequence to repair damaged DNA, HR requires strand invasion mediated by the recombinase RAD51. Earlier findings show that PARP1 is dispensable for HR. PARylation appears to have little direct effect on HR since HR is normal in PARP-depleted cells [53]. However, PARP1 has been associated with HR-mediated repair and reactivation of stalled replication forks, therefore promoting faithful DNA replication [56]. Moreover, PARP1 facilitates recruitment of MRE11 and RAD51, which restart stalled replication BRCA1/2-dependent early DDR [57]. The BRCTs of BARD1, the oligonucleotide/oligosaccharide binding-fold (OB-fold) of BRCA2, and the protein incorporated later into tight junctions (PilT) N terminus (PIN) domain of exonuclease 1 (EXO1) are the PAR-binding modules that target these HR repair machineries to DSBs for damaged DNA repair [58].

### PAR-binding modules

To regulate numerous biological functions, PAR chains must be recognized by diverse proteins such as DDR factors. To date, several distinct classes of PAR-binding modules have been identified. These include the PAR-binding zinc finger (PBZ), the Macro domain, the WWE domain, the BRCT domain, the forkhead-associated (FHA) domain, the OB-fold domain, the PIN domain, and the RNA recognition motif (RRM) domain [9], [59].

### PBZ domain

**Figure 2:**
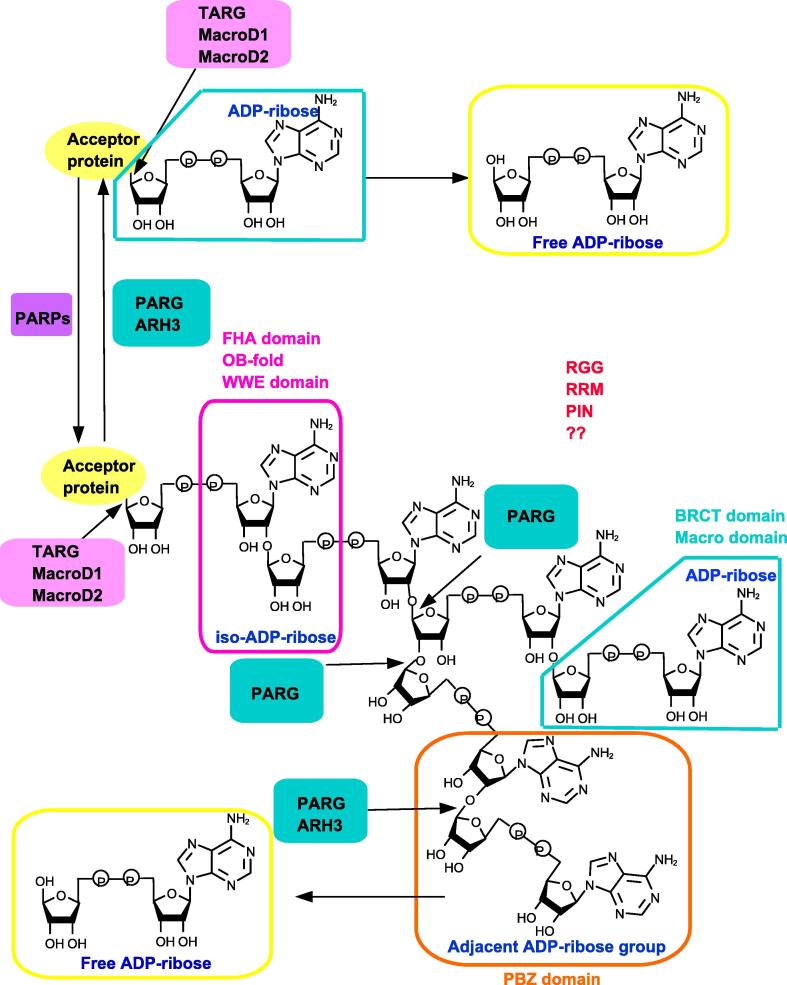
**PAR-binding modules** Using NAD^+^ as substrate, PARPs can produce PAR chain. For degradation, PARG and ARH3 mainly cleave the bonds of the PAR chain, except the proximal protein-bound ADP-ribose. The hydrolysis of last ADP-ribose from modified protein is conducted by TARG, MacroD1, and MacroD2. PBZ, WWE, Macro domain, FHA domain, OB-fold BRCT domain, RRM, and RGG motifs/domains can recognize the different parts of the PAR chain. ARH3, ADP-ribosylhydrolase 3; BRCT, BRCA1 C terminus; FHA, forkhead-associated; MacroD, Macro domain-containing protein; OB-fold, oligonucleotide/oligosaccharide-binding domain; PAR, poly(ADP ribose); PARG, PAR glycohydrolase; PARP, PAR polymerase; PBZ, PAR-binding zinc-finger; PIN, protein incorporated later into tight junctions (PilT) N terminus; RGG, arginine-glycine-glycine; RRM, RNA recognition motif; TARG, terminal ADP-ribose glycohydrolase; WWE, tryptophan-tryptophan-glutamate.

The recently-identified PBZ domains possess the consensus sequence [K/R]xxCx[F/Y]GxxCxbbxxxxHxxx[F/Y]xH [60]. PBZs are less common in mammalian proteins involved in DNA repair and cell cycle checkpoint, although PBZs are much more widespread in some other eukaryotes [48], [60], [61], [62]. Up till now, PBZ domains are only found in three human proteins, including histone chaperone APLF, checkpoint with FHA and RING finger (CHFR), and sensitive to nitrogen mustard 1A (SNM1A) [48], [60], [61], [62]. Crystal structures of APLF and CHFR show that PBZs are essential for their functions. Initial analysis of CHFR primary sequence has identified a zing finger called C2H2, which binds to PAR efficiently. Therefore, this motif is defined as a new PAR binding module termed PBZ [60]. APLF contains two tandem PBZ domains termed F1 and F2. Although F1 and F2 can recognize the PAR chain independently, presence of both domains remarkably increase the affinity of PAR chain binding, which is over 1000 times more efficient than the isolated PBZ domain [63]. Structural analysis demonstrates that PBZ module contains a central zinc ion coordinated by two cysteine and two histidine residues, which can recognize adenines in two neighboring ADP-ribose units of the PAR chain. This type of recognition renders the PBZ motifs to be the truly specific PAR binding modules [63] (Figure 2).

### The WWE domain

The WWE domain is the most recently discovered PAR-binding domain, named after the three strictly conserved amino acid residues, tryptophan-tryptophan-glutamate (WWE) [64]. The WWE domains, which can recognize iso-ADPR of PAR chain with high affinity, tightly links ubiquitination and PARylation signal pathways. The iso-ADPR which contains a characteristic bond, 2′,1″-*O*-glycosidic ribose-ribose is the signature of PAR chains [64]. The negatively-charged phosphate groups of the iso-ADPR can bind the positively-charged WWE domain [64]. The WWE domain is primarily found in two distinct protein families, including the E3 ubiquitin ligases (RNF146, deltex1, and TRIP12) and the PARPs (PARP8 and PARP11–14) [63]. So far, the function of WWE domain has been well described for RNF146/Iduna. RNF146 recognizes PAR chain and ubiquitinates DNA repair proteins such as XRCC1, PARP1, DNA ligase III, and Ku70. The PARylated proteins are targeted to proteosome for degradation [64], [65]. Taken together, the WWE domain-containing proteins are tightly linked with and influence each other (Figure 2).

### The Macro domain

The Macro domain, which consists of 130–190 amino acid residues, is evolutionarily conserved and widely spread throughout all kingdoms of organisms. This is distinct from the PBZ and WWE domains. It is estimated that more than 300 proteins, including 11 human proteins, with a diverse set of biological functions possess the Macro domain [66]. Macro domains can bind to the terminal ADPR of PAR, MAR, as well as *O*-acetyl-ADPR [66], [67], [68]. Some proteins such as amplified in liver cancer 1 (ALC1, also known as CHD1L), can interact with PAR chains through Macro domains and catalyze PARP1-stimulated nucleosome sliding, thus participating in DDR and chromatin remodeling [69], [70]. Some other Macro domain-containing proteins, in addition to their binding ability, also exhibit catalytic activity on the hydrolysis of PAR chains, making the Macro domains unique among the other PAR-binding modules. These include PARG [22], TARG1 [26], [71], and MacroD1/2 [28], [31] (Figure 2). PARG enzyme uses Macro domain for the binding and hydrolysis of PAR chains, as we outlined above.

### Additional domains

It is well known that FHA and BRCT domains can bind to phosphorylated proteins and modify protein–protein interactions [72]. Recently, it was reported that the phosphate-binding pocket in the central BRCT domain of BARD1 is required for selective binding to PAR chain [50], [73]. Meanwhile, BRCT domain promotes the interaction between BARD1 and PARP1. Moreover, the FHA domains of aprataxin (APTX) and PNKP confer affinity to iso-ADPR of PAR chain [50], [73].

The OB-fold is an ssDNA or ssRNA binding domain that has been found in proteins from all three kingdoms. OB-fold comprises 70–150 AA residues forming five-stranded beta-barrel with a terminating alpha-helix [57]. Interestingly, it is reported recently that the OB-fold can bind to the PAR-specific iso-ADPR and such binding is required to bring the ssDNA-binding protein 1 (SSB1) to sites of DNA damage [58].

The PIN domain-containing proteins serve as nucleases that cleave ssDNA/ssRNA in a sequence-specific manner [74]. The PIN domain consists of ∼130 amino acid residues characterized by a group of three strictly conserved acidic amino acid residues [75]. Our recent study found that the PIN domain of EXO1 recognizes PAR in DDR [58].

The RRM is one of the most abundant protein domains in eukaryotes, which can serve as a plastic RNA-binding platform to regulate post-transcriptional gene expression [76]. Several RRM-containing proteins have been reported to assemble at sites of PAR formation to promote DDR [77], [78].

It is reported that some RNA and DNA binding motifs can recognize PAR chains. Motifs enriched in arginines and glycines, which are termed glycine-arginine-rich (GAR) domains and/or RGG boxes, were identified several decades ago. RGG boxes are found in more than 1000 human proteins that are involved in numerous biological processes including transcription and DDR [79]. RGG boxes in the RNA-binding proteins such as FUS*/*TLS, EWS*/*EWSR1, TAF15, SAFB1, SAF-A, and hnRNPUL1*/*2, have been identified, and these proteins can be recruited to DNA damage sites via binding to PAR chain through RGG boxes [80], [81], [82], [83], [84], [85], [86].

### PARylation and ubiquitination

Ubiquitin is a small regulatory protein consisting of 76 amino acid residues, which has been found in almost all tissues of eukaryotic organisms. It can be covalently transferred to a Lys residue of an acceptor protein. This process is termed ubiquitination [87]. The ubiquitination pathway in cells is an elaborate system for targeting unwanted proteins for degradation, carried out by three classes of enzymes, E1, E2, and E3. Ubiquitin is first activated by ubiquitin-activating enzyme (E1) before being transferred to the active site of E1 in an ATP-dependent manner. Then the ubiquitin molecule is passed on to the second enzyme, ubiquitin-conjugating enzyme (E2), where ubiquitin is linked by another thioester bond to the Cys active site of E2. Finally, with the help of a third enzyme, ubiquitin protein ligase (E3), ubiquitin is transferred from E2 to a Lys residue on a substrate protein. Additional ubiquitin molecules can be linked to the first one to form a poly-ubiquitin chain usually targeting the protein to the proteasome [87].

Recent studies have demonstrated that PARylation can serve as a signal for the ubiquitination and promote the degradation of PARsylated proteins [88], [89], [90]. Some E3 ligases bind PAR via either a WWE (RNF146, also known as Iduna) domain or a PBZ (CHFR) domain [43], [44], [46]. The relationship between PARylation and ubiquitination has been well described in the RING-type E3 ubiquitin ligase, RNF146. The RNF146 WWE domain recognizes the PAR chain via interacting with iso-ADPR (Figure 2), functioning as an allosteric signal that changes the RING domain conformation from a catalytically-inactive state to an active one. RNF146 can polyubiquitylate many repair factors in a PAR-dependent manner, such as PARP-2, XRCC1, DNA ligase III, and Ku70 [62]. The discovery of a direct connection between PARylation and ubiquitination provides us with a new interpretation of the signaling function of PAR—degradation of proteins in a timely and orchestrated manner.

### Dysregulation of PARylation and human diseases

PARP1 is a key facilitator of DDR and is implicated in tumorigenesis of several malignancies, particularly those associated with dysfunctional DNA repair pathways [37]. Recent studies further demonstrate that transcript, protein, and enzyme activity of PARP1were increased in several tumor types with the most striking differences noticed in ovarian cancer, hepatocellular cancer, colorectal cancer, and leukemia [76], [77], [78]. Given that PARP1 has an important role in DDR, a novel therapeutic targeting PARP1 has been developed to treat cancers through increasing tumor sensitivity to chemotherapeutic agents and also through inducing “synthetic lethality” in cells [78]. Now PARP inhibitors have demonstrated efficacy in a number of tumor types, including platinum-sensitive epithelial ovarian cancer [50], breast cancer with mutation in BRCA1 or BRCA2 [91], and prostate cancer [92]. Olaparib is a PARP inhibitor that blocks enzymes involved in repairing damaged DNA [92]. Recently olaparib has been licensed as monotherapy for the treatment of patients with hereditary BRCA1 or BRCA2 mutations [91].

### Perspectives and conclusions

Over the last decades, PARylation has been proved to be involved in numerous cellular functions including DDR. PAR serves as an initial sensor and mediates the early recruitment of DNA damage repair machineries. As a kind of protein modification, PARylation is tightly and dynamically regulated. PAR chain synthesis is mediated by several PARPs, whereas PARG mainly takes charge of PAR chain degradation. Great strides have been made in the past few decades to decipher the PARylation regulatory processes and the underlying molecular mechanisms. However, many questions remain to be answered. First, other NAD^+^-consuming enzymes, such as sirtuin 1, are thought to compete for NAD^+^ with PARPs [9]. What is the reciprocal influence of these enzymes? Moreover, how these DNA damage factors are assembled at the DNA damage sites via PAR chains is still unclear exactly, as PAR chain does not have any sequence specificity. In addition, new molecular or chemical methods need to be developed to better achieve cell-permeable PARG or/and ARH inhibitors. More investigations are needed to address these questions in the future. In this regard, a better understanding of the biochemical and functional properties of PARylation in DNA repair may provide new clues to answer these fundamental questions.

## Competing interests

The authors declare that they have no competing financial interests.
